# Description of *Epidaus nanlingensis* sp. nov. (Hemiptera, Reduviidae, Harpactorinae) from Nanling Mountains, China, with an updated key to Chinese species of *Epidaus* Stål

**DOI:** 10.3897/BDJ.14.e189283

**Published:** 2026-04-21

**Authors:** Zhaoxiong Wang, Huaiyu Liu, Zhuo Chen, Chaorong Li, Xingmin Wang, Wanzhi Cai

**Affiliations:** 1 Department of Entomology, College of Plant Protection, South China Agricultural University, Guangzhou, China Department of Entomology, College of Plant Protection, South China Agricultural University Guangzhou China; 2 Engineering Research Center of Biological Control, Ministry of Education, Guangzhou, China Engineering Research Center of Biological Control, Ministry of Education Guangzhou China; 3 State Key Laboratory of Agricultural and Forestry Biosecurity, MARA Key Lab of Pest Monitoring and Green Management, College of Plant Protection, China Agricultural University, Beijing, China State Key Laboratory of Agricultural and Forestry Biosecurity, MARA Key Lab of Pest Monitoring and Green Management, College of Plant Protection, China Agricultural University Beijing China; 4 Administration of Guangdong Nanling National Nature Reserve, Shaoguan, China Administration of Guangdong Nanling National Nature Reserve Shaoguan China

**Keywords:** Heteroptera, Harpactorini, morphology, new species, Oriental Region

## Abstract

**Background:**

The assassin bug genus *Epidaus* Stål, 1859 (Hemiptera, Reduviidae, Harpactorinae) includes 28 species mainly distributed in the Oriental and Palaearctic Regions, with China being a major diversity hotspot, hosting nine known species.

**New information:**

*Epidaus nanlingensis* Wang Z, Liu & Cai, **sp. nov.** from Nanling National Nature Reserve, Guangdong, China, is described and illustrated, based on both male and female specimens. A comparative study of the new species and its morphologically similar species is presented. An updated identification key to all ten Chinese species of the genus *Epidaus* is provided.

## Introduction

The Nanling Mountain Area is a vast mountainous region located in southern China, spanning five provinces and accommodating 25 national Nature Reserves. This area has been recognised as a native biodiversity hotspot for endemic plants and vertebrates (Tang et al. 2006, Xu et al. 2016, Wang et al. 2024) and profoundly influences the evolution of the local biota (Wang and Dong 2018, Wang et al. 2023). However, studies on the insect fauna in the Nanling Mountain Area are still limited (Ge et al. 2024).

The assassin bug genus *Epidaus* Stål, 1859 (Hemiptera, Reduviidae, Harpactorinae, Harpactorini) currently contains 28 known species (Maldonado-Capriles 1990, Truong et al. 2006, Chen et al. 2016, Truong et al. 2025). The majority of these species are distributed in the Oriental and East Palaearctic Regions, with a single species, *E. pretiosus* Distant, 1903, being reported from New Guinea (Maldonado-Capriles 1990). China is a major diversity hotspot of *Epidaus*, with nine species being recorded in the country prior to this study (Yang 1940, China 1940, Hoffmann 1944, Hsiao 1979, Hsiao and Ren 1981, Putshkov and Putshkov 1996, Zhang et al. 2010, Chen et al. 2016).

The members of *Epidaus* can be recognised amongst Asian genera of Harpactorini by a combination of the following features: body elongate-oval; head slightly shorter than pronotum, with one process behind each antennal insertion; postocular region nearly 1.5 times as long as anteocular; first visible segment of labium as long as second and third segments combined; posterior lobe of pronotum with one pair of spines or tubercles at middle; humeral angles of pronotum produced to form sharp spines, each spine with one much smaller tooth-like tubercle behind; fore femora thicker than middle and hind femora, with the latter two nearly similarly thickened (Stål 1859, Hsiao and Ren 1981, Chen et al. 2016).

In the present study, we describe a new species, *E. nanlingensis* sp. nov. from Nanling Mountain Area in southern China. Morphological comparison is conducted amongst the new species and its morphologically similar species. A key is provided for the identification of the *Epidaus* species occurring in China. The new discovery highlights the need for further comprehensive investigations into the diversity of assassin bugs in southern China.

## Materials and methods

This study is based on specimens deposited in the Entomological Museum of China Agricultural University, Beijing, China (CAU). Male genitalia were soaked in hot 10% sodium hydroxide (NaOH) solution for approximately 15 minutes to remove soft tissue, rinsed in distilled water and dissected under a Motic binocular dissecting microscope. Dissected genitalia were placed in a small vial with glycerine and pinned under the corresponding specimen after observation. Photographs of external structures were all taken by a Canon R7 digital camera, docked with a Canon macro lens EF 100 mm (Canon Inc., Tokyo, Japan) and male genitalia were photographed using a Canon 5D Mark IV digital camera with an attached Canon macro lens MP–E 65 mm (Canon Inc., Tokyo, Japan). Helicon Focus 8.1.0 (Helicon Soft Ltd., Kiev, Ukraine) was used for image stacking; measurements were obtained using a calibrated micrometer. Morphological terminology mainly follows Weirauch (2008) and Chen et al. (2016).

## Taxon treatments

### Epidaus nanlingensis

Wang Z, Liu & Cai sp. nov.

AD4F55DD-E418-5E63-9DCE-3681AEE3CC30

4E142FBC-4356-4B97-9487-CB22B27F42C6

#### Materials

- **Type status:** Holotype. **Occurrence:** recordNumber: CAU; recordedBy: Zhaoxiong Wang; sex: male; lifeStage: adult; occurrenceID: 5575D156-8A15-549A-A1B5-FE87A3953E4B (Deposited in CAU).; **Location:** country: China; stateProvince: Guangdong; county: Ruyuan; locality: Nanling National Nature Reserve; verbatimElevation: 1050 m; verbatimCoordinates: 24.9260°N, 113.0217°E; **Event:** eventDate: 10-14 Jun 2024
- **Type status:** Paratype. **Occurrence:** recordNumber: CAU; recordedBy: Zhaoxiong Wang; sex: female; lifeStage: adult; occurrenceID: 7EC0E092-B1C3-5919-853B-D02E5725FA6D (Deposited in CAU).; **Location:** country: China; stateProvince: Guangdong; county: Ruyuan; locality: Nanling National Nature Reserve; verbatimElevation: 1050 m; verbatimCoordinates: 24.9260°N, 113.0217°E; **Event:** eventDate: 10-14 Jun 2024
- **Type status:** Paratype. **Occurrence:** recordNumber: CAU; recordedBy: Heyu Lv; sex: female; lifeStage: adult; occurrenceID: D1C03279-FBAA-5B70-A008-8BBB6F6D9E4E (Deposited in CAU).; **Location:** country: China; stateProvince: Guangdong; county: Ruyuan; locality: Nanling National Nature Reserve; verbatimElevation: 1457 m; verbatimCoordinates: 24.8973°N, 113.0190°E; **Event:** eventDate: 12 Jun 2025

#### Description

**Figure 1.**
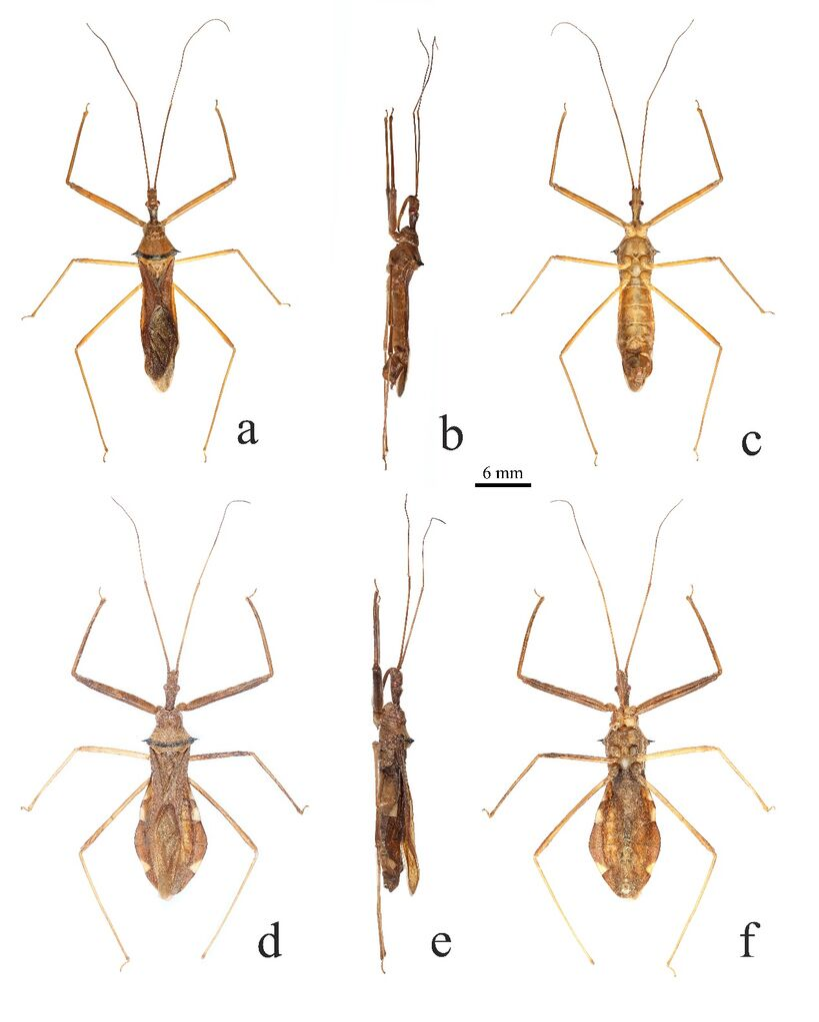
*Epidaus nanlingensis* sp. nov., habitus. **a-c** male, holotype; **d-f** female, paratype; **a, d** dorsal view; **b, e** lateral view; **c, f** ventral view.

**Figure 2.**
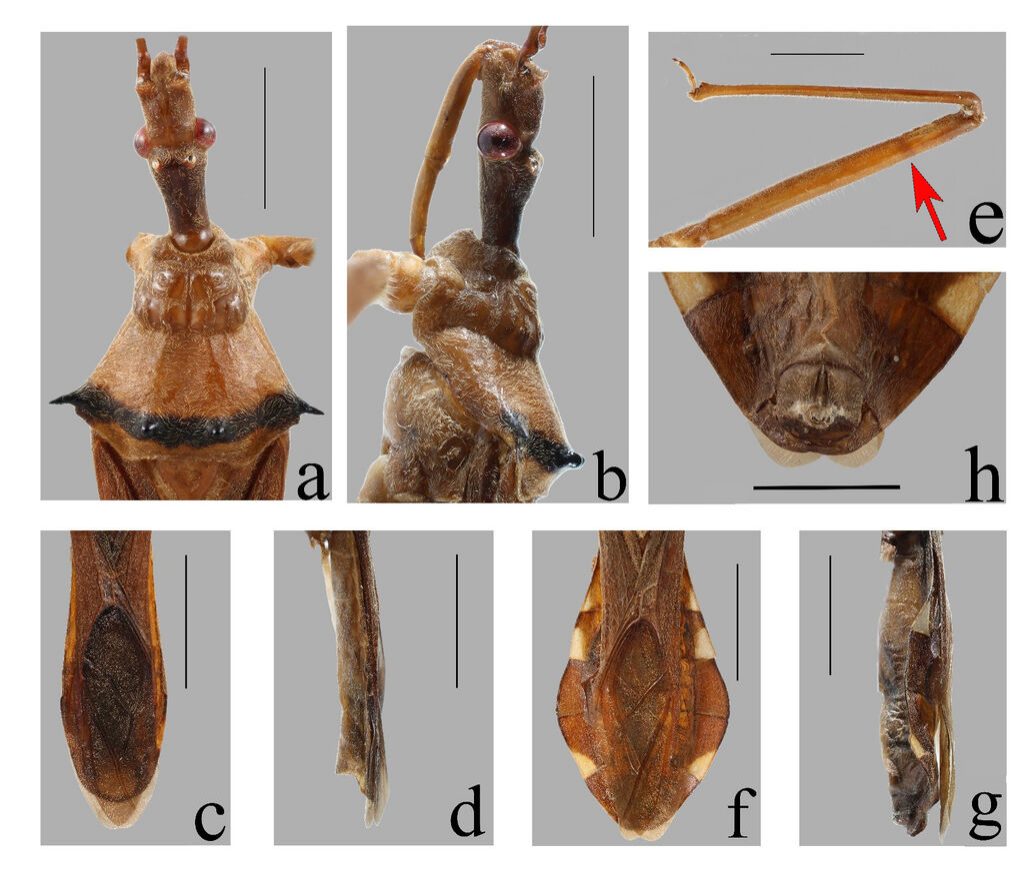
*Epidaus nanlingensis* sp. nov., male, holotype (**a-e**); female,paratype (**f-h**). **a, b** anterior part of body, with antennae and legs removed; **c, d, f-h** posterior part of body, legs not shown; **e** fore leg, the red arrow indicates the indistinct annulation on the fore femur. **a, c, e, f** dorsal view; **b, d, g** lateral view; **h** ventral view. Scale bars: a, b, e, h 3 mm; c, d, f, g 6 mm.

**Macropterous male: Colouration.** Generally yellowish-brown to brown (Fig. 1a-c). Head, antennae, eyes, pronotum, scutellum, hemelytra and connexivum (except the yellow markings) brown to reddish-brown; corium darker than membrane. Transverse band near posterior margin of posterior pronotal lobe, spines of humeral angles of pronotum, two tubercles in middle of posterior pronotal lobe black (Fig. 1a, b, Fig. 2a and b). Labium and legs yellowish-brown to light brown; indistinct annulation on subapical part of fore- and mid-femora brown (Fig. 1a, c, Fig. 2a, b and e). Apices of third, fourth and sixth connexival segments with very inconspicuous light yellow spots (Fig. 1a-c, Fig. 2c and d). Ventral surface of thorax and abdomen light brown (Fig. 1c).

**Vestiture.** Body clothed with short yellowish pubescence. Pubescence on pronotum and corium of hemelytra slightly longer and denser than that on pronotum and dorsal surface of abdomen (Fig. 1a). Lateral sides of anterior pronotal lobe with sparse, long, yellow setae amongst pubescence (Fig. 2a). Antennal segments sparsely covered with short setae (Fig. 1a-c). Ventral surface of fore trochanter and fore femora, inner surface of each tibia with dense thick setae; ventral surfaces of middle and hind femora with sparse setae (Fig. 1c and Fig. 2e). Dorsal surface of head with sparse, erect, blackish-brown setae interspersed amongst short pubescence; ventral surface of thorax and abdomen with sparse long setae interspersed amongst short pubescence (Fig. 2a and e).

**Structure.** Body medium-sized. Head subcylindrical; eyes medium-sized, protruding laterally; ocelli widely separated; synthlipsis wider than interocellar space; postocular region longer than anteocular; process behind antennal insertion blunt at apex. First antennal segment longest, longer than combined length of head and pronotum; fourth segment shortest (Fig. 1a-c). First visible segment of labium almost as long as second and third visible segments combined, exceeding posterior margin of eye; third segment shortest (Fig. 1b, c and Fig. 2b). Pronotum slightly shorter than its width; median longitudinal sulcus of anterior lobe deep at base; anterior lobe with sculptures, anterior margin concave; posterior lobe with one pair of coniform tubercles at middle (Fig. 1a, Fig. 2a and b); humeral angles spine-like, produced laterally; posterior margin nearly straight, posterior angles rounded and slightly protruding posteriorly. Scutellum triangular, basal width wider than median length, with Y-shaped carina (Fig. 1a). Fore femora slightly thickened, thicker than mid- and hind femora, claw of tarsi with small denticles (Fig. 2e). Hemelytra exceeding apex of abdomen; membrane slightly shiny, corium dull, without any wax spots. Abdomen with connexivum slightly expanded laterally (Fig. 1a-c, Fig. 2 c and d).

**Macropterous female**: Similar to male. Body generally reddish-brown; yellow spots on abdomen more conspicuous than those in male. Hemelytra shorter, slightly exceeding apex of abdomen; connexivum strongly expanded laterally (Fig. 1 d-f and Fig. 2 f-h).

**Figure 3.**
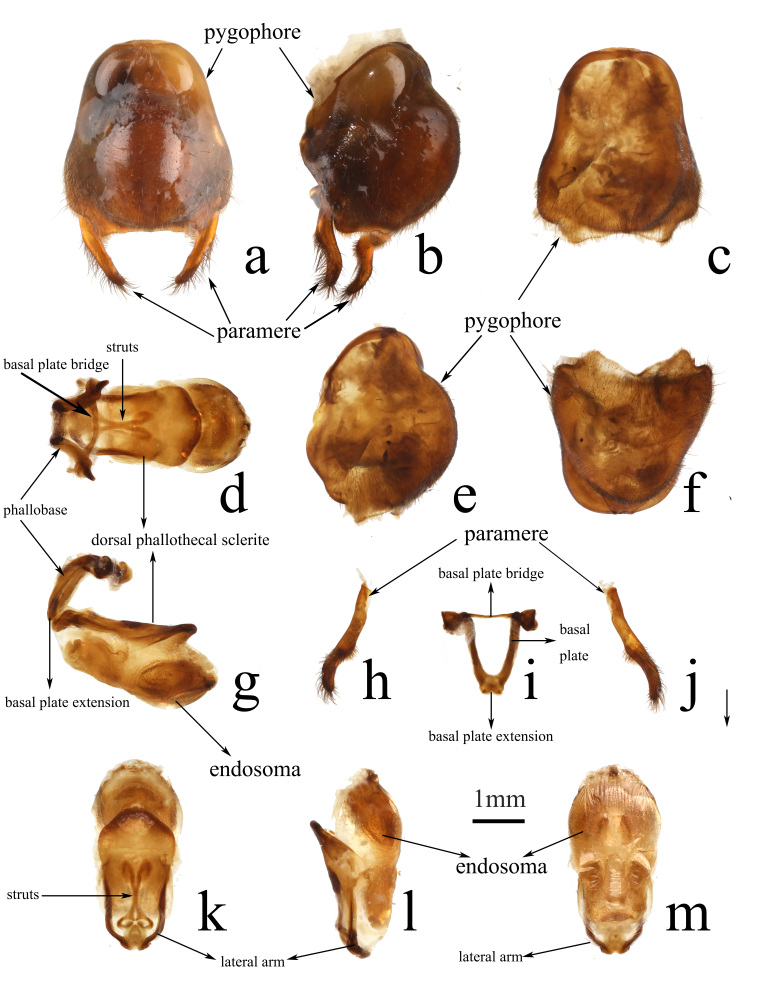
*Epidaus nanlingensis* sp. nov., male, holotype, genitalia. **a-c, e, f** pygophore; **h, j** paramere; **d, g** phallus **(whole)**; **i as phallobase**; **k-m as phallosoma**. **a, c, d, j, k** dorsal view; **b, e, g, l** lateral view; **h, m** ventral view. **f** caudal view.

**Male genitalia.** Pygophore oblong; median process wide, approximately three-fourths of maximum width of pygophore, concave at the middle, forming a triangular area on each side, slightly protruding laterally (Fig. 3a–c, e and f). Paramere clavate, slightly bent in basal half and strongly bent in apical half, blunt at apex, with abundant thick long setae on apical half (Fig. 3h and j). Basal plate thick, basal plate bridge thin, arched; basal plate extension short and wide (Fig. 3d, g and i). Phallosoma elongate oval (Fig. 3d, k and m); dorsal phallothecal sclerite warped upwards at apical part; struts strongly bent at base, sub-basal part slightly separated, fusing at middle, separating apically, highly sclerotised (Fig. 3d and k); lateral arm thick, highly sclerotised, arching slightly in lateral view (Fig. 3g and l). Distal dorsal lobe of endosoma slightly bulged, with two large bulges sparsely covered with tiny and small spines（Fig. 3g, k and l).

**Measurements** [male (n = 1) / female (n = 2), in mm]. Length of body: to apex of hemelytra 24.09 / 24.67–25.47, to apex of abdomen 22.50 / 24.36–25.31; length of head 4.28 / 4.76–4.83; length of anteocular region 1.65 /1.70–1.73; length of postocular region 2.36 / 2.40–2.64; synthlipsis 0.99 / 0.95–1.07; interocellar space 0.55 / 0.53–0.64; length of antennal segments I–IV = 9.90 / 10.30–10.72, 4.40 / 4.51–4.92, 7.32 / 6.49–7.17, 2.84 / ? (missing); length of anterior pronotal lobe 1.61 / 1.76–1.85; length of posterior pronotal lobe 2.67 / 2.56–2.76; width of pronotum 5.85 / 6.59–6.67; median length of scutellum 1.67 / 1.84–1.98; basal width of scutellum 1.79 / 2.10–2.22; length of hemelytron 15.71 / 15.79–17.70; length of visible labial segments I–III = 2.13 / 2.24–2.43, 1.56 / 1.52–1.66, 0.54 / 0.51–0.73.

#### Diagnosis

The new species can be recognised within the genus Epidaus by the following features: body medium-sized, generally yellowish-brown to reddish-brown; posterior lobe of pronotum with two black, coniform tubercles in middle and black transverse band near posterior margin (Fig. 1a, b, d, e, Fig. 2a and b); humeral angles of pronotum with black, long spines; connexivum of female roundly expanded laterally (Fig. 1d, f and Fig. 2f).

#### Etymology

**Figure 4.**
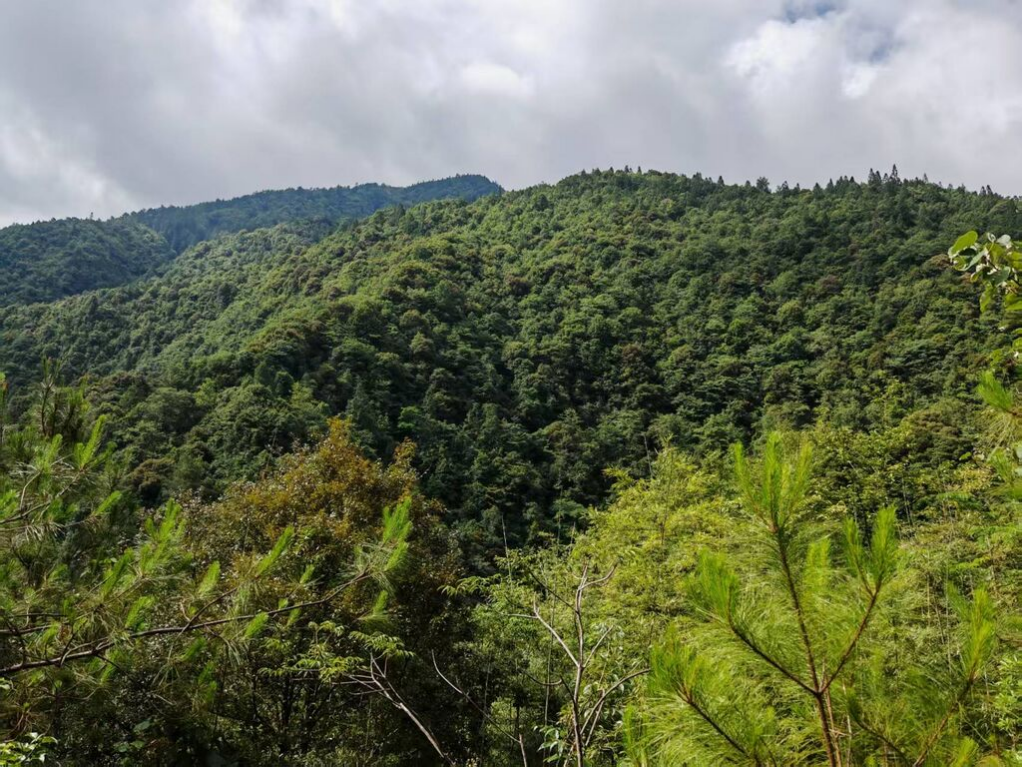
The type locality of *Epidaus nanlingensis* sp. nov. in Nanling National Nature Reserve, Guangdong, China.

The specific name alludes to the species’ type locality (Fig. 4), Nanling National Nature Reserve, Guangdong, China.

#### Distribution

China: Guangdong (Shaoguan).

## Identification Keys

### Key to the Chinese species of the genus Epidaus

**Table:** 

1	Pronotum with postero-lateral angles with short tubercles or short spines; median area of posterior pronotal lobe with two tubercles	2
–	Pronotum with postero-lateral angles with long spines; median area of posterior pronotal lobe with two tubercles or spines	3
2	Posterior margin of each connexival segment whitish-yellow	*E. nebulo* (Stål)
–	Fifth connexival segment totally reddish-brown in dorsal view; other segments pale-yellow	*E. tuberosus* Yang
3	Median area of posterior pronotal lobe with two spines	4
–	Median area of posterior pronotal lobe with two tubercles	9
4	Scutellum, except apical part black	*E. atrispinus* Distant
–	Scutellum not black	5
5	Postocular part of head blackish-brown	6
–	Postocular part of head not blackish-brown.	7
6	Pronotum and corium clothed with dense white wax spots; posterior pronotal lobe without black transverse band near posterior margin	*E. famulus* (Stål)
–	Pronotum and corium without wax spots; posterior pronotal lobe with black transverse band near posterior margin	*E. sexspinus* Hsiao
7	Head, pronotum, scutellum reddish-yellow	*E. bicolor* Distant
–	Head, pronotum (except transverse strip), scutellum pale yellow or brownish	8
8	Posterior pronotal lobe without black transverse band near posterior margin	*E. insularis* Zhang, Zhao, Cao & Cai
–	Posterior pronotal lobe with black transverse band near posterior margin	*E. longispinus* Hsiao
9	Body generally lemon yellow to yolk yellow; all femora with black annulations	*E. wangi* Chen, Zhu, Wang & Cai
–	Body generally yellowish-brown to reddish-brown; only fore- and mid-femora with blurred annulations	*E. nanlingensis* sp. nov.

## Discussion

The Nanling Mountains constitute the largest tectonic mountain belt in southern subtropical China, harbouring exceptionally high richness and endemism of biodiversity. Recent studies on the Coleoptera fauna of the Nanling Mountains have underscored the area’s significance as a critical biodiversity hotspot (Ge et al. 2024). However, research on the Heteroptera of the Nanling Mountains remains notably scarce.

The Nanling Mountains are the habitat of four known Epidaus species: *E. famulus* (Stål, 1863), 1979, *E. nebulo* (Stål, 1863), *E. sexspinus* Hsiao, 1979 and *E. tuberosus* Yang, 1940 (Li et al. 2023). Amongst them, the new species can be easily distinguished from *E. famulus* by the absence of the white wax spots on the pronotum and hemelytra, while sharing a similar general appearance with *E. nebulo*, *E. sexspinus* and *E. tuberosus*; the new species differs from *E. nebulo* (from China) by the different abdominal colour patterns, which has light yellowish markings only on the apical half of the third, fourth and sixth connexival segments (vs. light yellowish marking present on all connexival segments in E. nebulo) and the long spine-like humeral angles of the pronotum (vs. short conical in E. nebulo); the new species can also be distinguished from *E. sexspinus* (from China, Vietnam and Japan) by having two tubercles at the middle of the posterior lobe of the pronotum (vs. with two spines in E. sexspinus), more expanded fifth and sixth connexival segments and the different colour patterns of the abdomen (light yellowish markings are restricted to the apical half of specific segments vs. light yellowish spots present on the second to sixth segments in E. sexspinus); also, the new species can be separated from *E. tuberosus* (from China, the Korean Peninsula and Russia) by having long spine-like humeral angles of the pronotum (vs. short conical in E. tuberosus), the presence of a black transverse band on the pronotum (vs. lacking such band in E. tuberosus) and the different colour patterns of the abdomen.

The new species can also be clearly distinguished from some representative *Epidaus* species occurring outside China. For instance, the new species differs from *E. bachmaensis* Truong, Zhao & Cai, 2006 (from Vietnam) and *E. validispinus* Stål, 1863 (from Borneo) in the pronotum not covered with dense waxy bloom or long pubescence, with the median pronotal elevation tuberculate (vs. developed into a strong spine as in *E. bachmaensis* and *E. validispinus*), along with the reddish-brown connexivum with yellowish-white spots (vs. the mostly yellowish-white connexivum with three reddish-yellow spots of *E. bachmaensis* or the brownish-orange unicoloured connexivum of *E. validispinus*); when comparing with *E. conspersus* Stål, 1863 (from India), *E. furculatus* Stål, 1863 (from Borneo) and *E. maculiger* Stål, 1859 (from Leyte Island, Philippines), the dark blackish-brown body colouration of these species is distinctly different from the reddish- or yellowish-brown body colour of the new species. The new species can be separated from *E. latispinus* Stål, 1863 (from Borneo) by the median posterior pronotal lobe with two tubercles (vs. denticle-like spines of *E. latispinus*); also, the new species lacks the white waxy spots on the hemelytra that are present in *E. latispinus*; the new species can also be easily distinguished from *E. batxatensis* Truong, Nguyen & Ha, 2025 and *E. konkakinhensis* Truong, Nguyen & Ha, 2025 (both from Vietnam) by several distinctly different features: apex of process behind antennal insertion of *E. batxatensis* is sharp and *E. batxatensis* possesses a bright yellowish body colour, instead of the blunt apex of the process behind antennal insertion and brownish body colour in the new species; *Epidaus konkakinhensis* differs from the new species by its well-developed, cone-like collar process and its pale colouration.

## Supplementary Material

XML Treatment for Epidaus nanlingensis
